# MMP-9 Serum Levels in Schizophrenic Patients during Treatment Augmentation with Sarcosine (Results of the PULSAR Study)

**DOI:** 10.3390/ijms17071075

**Published:** 2016-07-09

**Authors:** Dominik Strzelecki, Olga Kałużyńska, Justyna Szyburska, Adam Wysokiński

**Affiliations:** 1 Department of Affective and Psychotic Disorders, Medical University of Łódź, Central Clinical Hospital, Czechosłowacka 8/10, 92-216 Łódź, Poland; okaluzynska@gmail.com (O.K.); szyburska@gmail.com (J.S.); 2 Department of Old Age Psychiatry and Psychotic Disorders, Medical University of Łódź, Central Clinical Hospital, Czechosłowacka 8/10, 92-216 Łódź, Poland; adam.wysokinski@gmail.com

**Keywords:** matrix metallopeptidase-9 (MMP-9), sarcosine, NMDA (*N*-methyl-d-aspartate) receptor, glutamatergic system, schizophrenia, negative symptoms

## Abstract

Aim: Find changes in matrix metallopeptidase-9 (MMP-9) levels during augmentation of antipsychotic treatment with sarcosine and a relationship between schizophrenia symptoms severity and initial level of MMP-9. Method: Fifty-eight patients with diagnosis of schizophrenia with predominant negative symptoms participated in a six-month prospective RCT (randomized controlled trial). The patients received two grams of sarcosine (*n* = 28) or placebo (*n* = 30) daily. At the beginning, after six weeks and after six months MMP-9 levels were measured. Severity of symptomatology was assessed with the Positive and Negative Syndrome Scale (PANSS) and Calgary Depression Scale for Schizophrenia (CDSS). Results: MMP-9 serum levels were stable after six weeks and six months in both groups. We noted improvement in negative symptoms, general psychopathology and total PANSS score in sarcosine group compared to placebo; however, there was no correlations between serum MMP-9 concentrations and PANSS scores in all assessments. Initial serum MMP-9 concentrations cannot be used as an improvement predictor acquired during sarcosine augmentation. Conclusions: Our results indicate that either MMP-9 is not involved in the *N*-methyl-d-aspartate (NMDA)-dependent mechanism of sarcosine action in terms of clinical parameters or sarcosine induced changes in peripheral MMP-9 concentrations cannot be detected in blood assessments.

## 1. Introduction

### 1.1. Glutamate, NMDA (N-Methyl-d-aspartate) Receptor and Sarcosine

Glutamic acid (glutamate) is an amino acid serving as a neurotransmitter in the major excitatory system of the human brain—glutamatergic system. Fifty percent of brain neurons belong to this system, which, along with inhibitory GABAergic (γ-aminobutyric acid) system, is responsible for transmitting information and modulation of other system functioning. Glutamate with diverse population of its receptors—e.g., NMDA (*N*-methyl-d-aspartate) and AMPA (α-amino-3-hydroxy-5-methyl-4-isoxazolepropionic acid)—is involved in the pathogenesis of schizophrenia [1,2]. Antagonists of NMDA receptor such as phencyclidine (commonly known as “angel dust”) or ketamine cause symptoms mimicking schizophrenia [3] and exacerbate psychosis in schizophrenic patients including also negative and cognitive symptomatology [4]. Glycine is natural co-agonist of NMDA receptor and along with glutamate is required to activate ion flows through the receptor. Literature on the use of co-agonists of NMDA receptor (glycine, or similar substances such as d-serine, d-cycloserine and d-alanine) and glycine transporter inhibitors GlyT-1 (sarcosine) is now quite large in schizophrenia [5,6,7,8]. Astrocytes with glycine transport system (GlyT-1) are responsible for appropriate glycine levels in the synaptic cleft [9]. Sarcosine (*N*-methylglycine), having properties of glycine transporter system inhibitor in typical NMDA receptor localizations, increases levels of glycine and positively influences function of NMDA receptor (hypoNMDA hypothesis postulated in schizophrenia) [1,9]. The majority of studies suggests mild to moderate beneficial effects of these substances used as augmentation of antipsychotic drugs, particularly on negative and cognitive symptoms.

### 1.2. MMP-9 (Matrix Metallopeptidase-9), Schizophrenia and Other Psychiatric Disorders

Matrix metalloproteinase 9 (MMP-9, 92 kDa type IV collagenase, 92 kDa gelatinase or gelatinase B (GELB)) is pericellularly acting endopeptidase that play a key role in remodeling of the extracellular matrix by cutting extracellular substrates, including precursors of growth factors, cell receptors or adhesive molecules [10,11,12,13,14]. The group of metalloproteinases is involved in synaptic plasticity and long term potentiation (LTP) and thereby in memory processes [15,16,17,18,19], similarly as glutamatergic receptors. Furthermore, LTP secondarily increases MMP-9 activity. MMP-9 causes expansion of spines and synaptic potentiation [19]. In hippocampal cells and slices, exogenous MMP-9 promotes spines enlargement [19] or elongation [20,21]. These data indicate a novel, MMP-dependent, mechanism of the synaptic plasticity, however the exact impact of MMP-9 on synaptic remodeling is currently unclear.

Abnormalities of morphology of dendritic protrusions have been frequently described in neuropsychiatric disorders, particularly with deficits in cognition [22,23,24]. Recent data suggest involvement of MMP-9 in disturbed synaptic plasticity and morphology of spines in schizophrenia, bipolar disorder and alcohol dependency [25,26,27,28,29]. There is a need for further studies on specific, mechanistic role of MMP-9 in the neuropsychiatric disorders, which might help to develop new groups of psychiatric drugs. One possible mechanism links MMP-9 with Brain Derived Neurotrophic Factor (BDNF)—MMP-9 plays a key role in the conversion of proBDNF to mature BDNF. Genetic studies also indicate involvement of MMP-9 in pathogenesis of schizophrenia [30]. Recent reports, particularly important from the perspective of our study, associates activity of MMP-9 with function and localization of glutamate receptors [31,32,33].

### 1.3. MMP-9, Cardiometabolic and Metabolic Parameters

MMP-9 levels may be affected by cardiometabolic parameters (such as diabetes, hyperlipidemia and obesity) [34]. We assessed several biochemical (blood glucose, cholesterol and triglycerides), anthropometric (body weight and abdominal circumference) and cardiometabolic parameters. For best accuracy (since simple measurements, such as body mass index, may not reflect general or central obesity), we also performed body composition analysis, which provides accurate measurements of body fat and lean (fat-free) body mass. This is the first study to investigate a combination of biochemical, anthropometric and body composition parameters in subjects with schizophrenia. Considering the involvement of both MMP-9 and glutamate in synaptic plasticity, memory and other cognitive processes impaired in schizophrenia, we hypothesize the relationship between MMP-9 levels and psychopathology changes induced by sarcosine. The primary aim of our study was to assess MMP-9 changes as a result of sarcosine use. The secondary aim was to investigate whether MMP-9 levels correlate with severity of schizophrenic (assessed with the Positive and Negative Syndrome Scale (PANSS)) and depressive (assessed with the Calgary Depression Scale for Schizophrenia (CDSS)) symptoms and clinical improvement during our study.

## 2. Results

Sixty subjects were randomized to receive either sarcosine (*n* = 30) or matching placebo (*n* = 30) and completed a six-month, double blind, placebo-controlled study. Two patients in the sarcosine group did not complete the blood tests. Therefore, 58 schizophrenic patients, including 28 patients taking sarcosine, were subjected to the analysis. Apart from the percentage of smokers, as shown in the Table 1 and Table 2, groups were very comparable in terms of demographic, clinical, therapeutic, anthropometric and metabolic parameters. All participants remained on their antipsychotic treatment for the duration of the study.

Clinically, there were no differences between initial PANSS scores in both subgroups. However, patients from the sarcosine group showed significantly better improvement in PANSS scores at the end of the study compared with the placebo group (reduction in total score: −13.7 ± 8.7 vs. −2.3 ± 8.7, *p* < 0.001; reduction in negative subscore: −6.8 ± 3.5 vs. −0.7 ± 1.7, *p* < 0.001; reduction in general subscore: −6.7 ± 6.3 vs. −1.0 ± 5.3, *p* < 0.001). There was a significant reduction in CDSS score in the sarcosine group (sarcosine: −0.5 ± 2.1, placebo: 1.1 ± 3.2, *p* = 0.02). At the end of the study, there were no significant changes in any of the analyzed cardiometabolic and body composition parameters in both groups.

**Figure 1:**
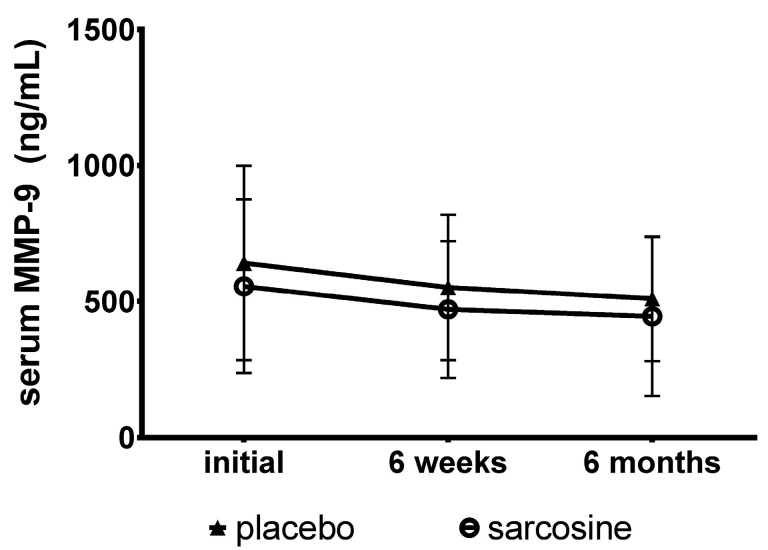
Mean MMP-9 (matrix metallopeptidase-9) levels in the study groups. Vertical bars represent standard deviations.

Figure 1 shows MMP-9 levels in the study groups in three times points (initial, after six weeks and after six months). There were no differences for MMP-9 levels between the study groups at the beginning of the study (sarcosine: 556.13 ± 318.89 ng/mL, placebo: 641.94 ± 357.42 ng/mL, *p* = 0.28), after six weeks (sarcosine: 470.74 ± 251.86 ng/mL, placebo: 551.95 ± 267.15 ng/mL, *p* = 0.37) and after six months (sarcosine: 445.13 ± 291.80 ng/mL, placebo: 510.15 ± 229.35 ng/mL, *p* = 0.20). Moreover, changes in MMP-9 levels did not differ significantly between the study groups after six weeks (sarcosine: −153.61 ± 227.38 ng/mL, placebo: −78.40 ± 287.13 ng/mL, *p* = 0.34) and after six months (sarcosine: −110.85 ± 376.54 ng/mL, placebo: −102.20 ± 317.70 ng/mL, *p* = 0.98). There were no statistically significant differences for changes in MMP-9 levels between depressed and non-depressed patients (sarcosine: −335.26 ± 542.39 ng/mL vs. −59.85 ± 323.72 ng/mL, *p* = 0.13; placebo: −106.18 ± 356.64 ng/mL vs. −101.56 ± 319.15 ng/mL, *p* = 0.45).

At the beginning of the study, there were 11 patients classified as depressed (sarcosine: 6, placebo: 5). Seven patients (sarcosine: 1, placebo: 6) were classified as depressed at the end of the study that were not classified as depressed at the beginning of the study, while five patients (sarcosine: 2, placebo: 3) were classified as non-depressed at the end of the study that were classified as depressed at the beginning of the study. The difference for these proportions was not significant. We compared changes in MMP-9 levels between subjects who become depressed or non-depressed at the end of the study with those who did not change their category and found no significant differences between these subgroups.

In the total study group and in both subgroups, we found that initial MMP-9 levels are correlated with changes in MMP-9 levels after six weeks (total study group: *r* = −0.70, *p* < 0.001; placebo: *r* = −0.74, *p* < 0.001; sarcosine: *r* = −0.69, *p* < 0.001) and after six months (total study group: *r* = −0.69, *p* < 0.001; placebo: *r* = −0.74, *p* < 0.001; sarcosine: *r* = −0.66, *p* < 0.001). We found no correlations between MMP-9 levels (total, deltas) and PANSS or CDSS scores (total, subscores or deltas).

There were no differences in initial MMP-9 levels between smokers and non-smokers in the whole study sample (*p* = 0.52), in the placebo group (*p* = 0.77) and in the sarcosine group (*p* = 0.47). There were also no differences for six-month change of MMP-9 levels between smokers and non-smokers in the whole study sample (*p* = 0.58), in the placebo group (*p* = 0.41) and in the sarcosine group (*p* = 0.84).

## 3. Discussion

Despite established involvement of MMP-9 in basic cognitive processes, the literature concerning the potential reciprocal relation between this metalloproteinase and schizophrenia is very sparse. MMP-9 concentrations in patients diagnosed with schizophrenia are higher than in healthy controls [25,35,36] in most of published research, while in a few studies significant differences were not observed [37,38]. In drug-free patients with schizophrenia, MMP-9 levels were higher comparing to controls; moreover, previous use of antipsychotics was associated with higher concentrations of MMP-9 [39]. In groups with resistant schizophrenia with ongoing clozapine treatment, levels of MMP-9 were higher comparing to healthy controls [36]. Further analysis in both studies showed no correlation of the severity of particular clinical parameters with MMP-9 levels. There are no published results of studies on the effects of antipsychotics on MMP-9 concentrations.

Our project is the first randomized placebo-controlled study to investigate the sarcosine (or glutamatergic agent in general) effects on MMP-9 serum levels in individuals with schizophrenia. We showed here that add-on treatment with sarcosine does not significantly change peripheral MMP-9 levels after six weeks and after six months. Assuming that MMP-9 concentrations may vary over time under sarcosine influence and after several weeks, its levels may be different than after long-term sarcosine augmentation, and this study was conducted in such a way to verify it. We did not find changes either after six weeks or after six months of sarcosine supplementation. Moreover, concentrations of MMP-9 between two our groups also did not differ significantly at any of three time points. Since our study subgroups were very homogenous in terms of clinical, anthropometric or cardio-metabolic parameters that may possibly affect levels of MMP-9, we assumed that the observed lack of differences cannot be attributed to these variables.

Antidepressive treatment may also influence expression of MMP-9 mRNA [40]. However, both of our groups did not significantly differ in their antidepressive treatment. Antipsychotic treatment in our patients was heterogeneous, but comparison of defined daily doses (DDD, see [App app1-ijms-17-01075]Appendix Table A1) were statistically comparable in sarcosine and placebo group. Although in patients taking sarcosine we observed significant clinical improvement during the study, serum MMP-9 levels remained stable as in placebo group, where clinical parameters remained unchanged during the study. As a result, we failed to reveal a correlation between peripheral MMP-9 levels and PANSS total score, its subscores (positive symptoms, negative symptoms and general psychopathology) or CDSS score in different time-points. Observations concerning both subgroups and the whole study group show higher increase of MMP-9 concentrations in patients with initially lower levels of the metalloproteinase in subsequent measurements.

Smoking status can influence the levels of MMP-9 [37], but, according to comparisons in sarcosine and placebo groups, there was no significant impact of tobacco use on initial and subsequent metalloproteinase concentrations.

We failed to prove that there is a mechanism induced by sarcosine that could change serum MMP-9 levels. Therefore, we conclude that there is need for further studies, in which assessment of MMP-9 levels will be performed in particular brain regions particularly important in schizophrenia, such as dorsolateral prefrontal cortex, anterior cingulate cortex or hippocampus. Because of the involvement of MMP-9 in cognitive processes, it would also be very interesting to assess the impact of sarcosine on working memory, attention, visual-spatial skills and other aspects of cognition and to correlate these parameters with MMP-9 concentrations. The role of metalloproteinase 9 in affective disorders (depression and bipolar disorders) is currently investigated, indicating association between MMP-9 level, depression severity and treatment [38]. Although depressive syndromes occurred in our patients, the two groups did not differ significantly in terms of frequency and severity of these disorders and MMP-9 levels.

Because of the lack of changes in serum MMP-9 levels and a relatively small group size, an analysis regarding the potential prognostic value of the baseline MMP-9 concentration was considered pointless.

## 4. Materials and Methods

### 4.1. Participants and Study Design

Study participants aged 18–60 years with diagnosis of paranoid schizophrenia (295.30, according to DSM-IV (Diagnostic and Statistical Manual of Mental Disorders, 4th Edition), F20.0 according to ICD-10 (10th revision of the International Statistical Classification of Diseases and Related Health Problems)). Recrutation was conducted in outpatient clinics. Patients in stable physical, neurological and endocrinological condition with normal laboratory values (electrocardiogram, blood tests, biochemical tests including, liver, kidney and thyroid glandule parameters) were enrolled to the study. Before all procedures patients were examined with structured interview according to ICD-10 and DSM-IV criteria of schizophrenia. Clinical inclusion criteria included (1) scoring a minimum of 3 points in each item in negative symptoms subscale and a maximum of 3 points in positive symptoms subscale of the PANSS; (2) stable mental state and stable treatment for at least three months before signing the informed consent form. Among the criteria for exclusion were acute psychotic state, suicidal tendencies and treatment with clozapine, because combination of clozapine with glutamatergic drugs may impair mental condition by increasing both positive and negative symptomatology [41,42].

Our project was six months randomized, double blind, placebo-controlled and parallel group study. Patients were randomly (1:1 ratio) assigned to sarcosine or placebo group. Subjects received Eppendorf tubes containing two grams of sarcosine or placebo (microcrystalline cellulose). Patients in both groups were advised to dissolve and drink powder from capsule once a day after breakfast.

All enrolled patients signed informed consent form for participation in this study after receiving comprehensive information about aims and methods of our project. More details of Polish Sarcosine Study in Schizophrenia (PULSAR) we provide in acknowledgments.

#### Treatment

In our study, treatment with first- and second-generation antipsychotics and antidepressants (selective serotonin or serotonin and noradrenaline reuptake inhibitors, tricyclic antidepressant clomipramine) was heterogeneous. Detailed information about the treatment (medications and doses) is shown in the Appendix.

### 4.2. Measurements

All measurements were taken twice—during the first study visit and during the last study visit, after six months. Moreover, in 13 patients from the placebo group and in 15 patients from the sarcosine group, additional MMP-9 tests were performed after six weeks.

#### 4.2.1. Clinical Assessments

Clinical symptoms of schizophrenia were assessed using PANSS scale and its subscores (positive, negative and general symptoms) [43], and severity of depression was assessed using the Calgary Depression Scale for Schizophrenia (CDSS) [44]. For each patient, assessments with all scales were performed by one trained rater. Patients were defined as depressed when CDSS was >6.

#### 4.2.2. Blood

The blood samples were collected in the morning, after ensuring at least 8 h of overnight fasting. Immediately after collection, blood samples were centrifuged (3500 rpm at 22 °C for 10 min) and analyzed. Serum glucose and lipids levels were measured using a Dirui CS-400 analyzer (Dirui, Changchun, China). Levels of MMP-9 were measured in duplicate in serum samples using ELISA (enzyme-linked immunosorbent assay) method, with a commercial human MMP-9 kit (R & D Systems, Minneapolis, MN, USA), intra-assay Coefficient of Variability (CV) < 8.4%, inter-assay CV < 9.6%, detection sensitivity ranged from 0.003 to 0.014 ng/mL. The mean MDD (minimum detectable dose) was 0.007 ng/mL. Prior to ELISA assessments serum samples were stored at −80 °C. Protocols were performed according to the producer’s instructions. The optical density of wells was measured using an automated microplate reader (Emax; Molecular Devices, Sunnyvale, CA, USA).

#### 4.2.3. Anthropometry

Height was measured with a wall-mounted height measure to the nearest 0.5 cm. Weight was measured to the nearest 0.5 kg with a spring balance, with subjects wearing light clothing, without shoes. Body mass index (BMI) was calculated as body weight (kg)/height (m^2^). Waist, abdominal and hip circumferences were measured using a non-stretchable fiber measuring tape at standard levels.

#### 4.2.4. Body Composition

We used the method of bioelectrical impedance analysis (BIA) for the assessment of body composition (body fat and lean body mass). Briefly, by measuring the flow of a weak electric current through body tissues, BIA determines the electrical impedance to calculate total body water. This can be used to estimate lean (fat-free) body mass and, by difference with body weight, body fat. For BIA measurements, we used Maltron BF-906 Body Fat Analyzer (Maltron, Rayleigh, UK), single frequency (50 Hz) bioelectrical impedance analyzer. Body composition was measured in standard conditions (participants lying supine, resting), by a trained technician, immediately prior to anthropometry measurements.

#### 4.2.5. Determination of Metabolic Syndrome and Other Measurements

International Diabetes Foundation (IDF) criteria were used to define metabolic syndrome and abdominal obesity [45]. Fasting plasma glucose ≥100 mg/dL was defined as impaired fasting glucose. Dyslipidemia was defined as triglycerides (TGA) ≥150 mg/dL and/or total cholesterol (TC) ≥200 mg/dL and/or high-density lipoproteins (HDL) cholesterol <40 mg/dL for men and <50 mg/dL for women and/or low-density lipoproteins (LDL) cholesterol ≥135 mg/dL. Waist-to-hip ratio (WHR) was calculated as waist circumference divided by hip circumference. Fat mass index (FMI) was calculated as total body fat in kilogram divided by the height in meter squared (kg/m^2^).

### 4.3. Statistical Analysis

The randomization code was not broken until all data were collected. Statistical analysis was performed using STATA 14.1 (StataCorp, College Station, TX, USA). For continuous variables, descriptive statistics (means, standard deviations, and 95% confidence interval (CI)) were calculated, while for discrete variables number of patients and percentages are given. Shapiro–Wilk test was used to test normality of distribution, and variables that did not follow normal distribution were transformed for best normality. Means, standard deviations, and confidence intervals are reported for non-transformed variables, results of tests are reported for transformed or non-transformed variables. The difference between proportions was analyzed by Fisher’s exact test, while inter-group differences were assessed by *t*-test. *p* values less than 0.05 were considered as significant (two sided).

## 5. Limitation of the Study

The main limitations of the study are:

1. relatively small study groups;
2. lack of a healthy control group;
3. heterogeneous antipsychotic treatment across the study groups; and
4. groups including only patients with dominant negative symptoms.

## 6. Conclusions

After six weeks and six months of our study, serum levels of MMP-9 were stable in both groups. We observed improvement in negative symptoms, general psychopathology and total PANSS score in sarcosine group compared to placebo group, but there was no correlations between serum MMP-9 concentrations and PANSS scores in all assessments.

Initial serum MMP-9 concentrations cannot be used as a predictor of the improvement resulting from sarcosine addition in patients with schizophrenia in stable mental condition.

## Figures and Tables

**Table 1:** Baseline participant characteristics.

Parameter	Sarcosine (*n* = 28)	Placebo (*n* = 30)	*p*
Men	19 (67.9%)	15 (50.0%)	NS
Age (years)	38.0 ± 11.0 (33.7–42.2)	40.2 ± 10.1 (36.4–44.0)	NS
Education (years)	14.4 ± 2.7 (13.4–15.4)	14.0 ± 2.6 (13.0–14.9)	NS
Smoking	9 (32.1%)	19 (63.3%)	0.02
Treatment duration (years)	14.6 ± 8.9 (11.2–18.0)	11.6 ± 4.9 (9.7–13.4)	NS
Number of hospitalizations	4.8 ± 5.7 (2.5–7.0)	4.2 ± 4.8 (2.4–5.9)	NS
Time from last hospitalization (years)	2.9 ± 4.2 (1.3–4.6)	4.7 ± 4.6 (2.9–6.5)	NS
SGA	24 (88.9%)	28 (96.5%)	NS
FGA	4 (14.8%)	8 (26.7%)	NS
Antidepressants	12 (44.4%)	11 (36.7%)	NS
Initial PANSS score	69.5 ± 14.2 (64.0–75.0)	72.5 ± 12.5 (67.8–77.1)	NS
Positive subscale	10.2 ± 2.9 (9.0–11.3)	10.4 ± 3.1 (9.2–11.6)	NS
Negative subscale	25.7 ± 5.3 (23.6–27.7)	26.1 ± 5.0 (24.3–28.0)	NS
General subscale	33.6 ± 8.2 (30.5–36.8)	35.9 ± 7.4 (33.1–38.7)	NS
Initial CDSS score	3.7 ± 3.1 (2.5–4.9)	3.6 ± 2.8 (2.5–4.6)	NS
Patients with depression	6 (21.4%)	5 (16.7%)	NS

Data given as: *n* (%) or mean ± standard deviation (95% CI (confidential intervals)); SGA = second-generation antipsychotics; FGA = first-generation antipsychotics; PANSS = Positive and Negative Syndrome Scale; CDSS = Calgary Depression Scale for Schizophrenia; NS = non significant.

**Table 2:** Cardio-metabolic characteristics.

Parameter	Sarcosine (*n* = 27)	Placebo (*n* = 30)	*p*
SBP (mm Hg)	123.6 ± 16.3 (117.2–129.9)	126.7 ± 16.4 (120.6–132.8)	NS
DBP (mm Hg)	74.4 ± 9.2 (70.8–78.0)	79.3 ± 9.2 (75.9–82.7)	NS
TC (mg/dL)	202.8 ± 31.9 (190.4–215.1)	221.2 ± 54.1 (201.0–241.4)	NS
HDL (mg/dL)	45.4 ± 18.1 (38.4–52.4)	45.5 ± 14.8 (40.0–51.1)	NS
LDL (mg/dL)	126.0 ± 27.5 (115.3–136.6)	143.6 ± 43.9 (127.6–160.2)	NS
TGA (mg/dL)	155.6 ± 77.8 (125.4–185.8)	161.3 ± 106.8 (121.4–201.2)	NS
FPG (mg/dL)	96.7 ± 14.3 (91.2–102.3)	97.6 ± 22.9 (89.0–106.1)	NS
TSH (μIU/mL)	1.7 ± 0.9 (1.3–2.0)	1.5 ± 0.7 (1.3–1.8)	NS
PRL (ng/mL)	33.8 ± 31.3 (21.7–45.9)	31.5 ± 36.6 (17.9–45.1)	NS
Antihypertensive treatment	4 (14.8%)	7 (23.3%)	NS
Lipid-lowering treatment	1 (3.7%)	2 (6.7%)	NS
Antidiabetic treatment	0	1 (3.7%)	NS
Metabolic syndrome	13 (48.1%)	18 (60.0%)	NS
Dyslipidemia	22 (81.5%)	25 (83.3%)	NS
Impaired fasting glucose	8 (29.6%)	9 (30.0%)	NS
Body Composition			
Weight (kg)	90.4 ± 21.1 (82.1–98.8)	86.4 ± 16.3 (80.3–92.5)	NS
BMI (kg/m^2^)	34.1 ± 22.5 (25.2–43.0)	29.4 ± 4.9 (27.6–31.3)	NS
FMI (kg/m^2^)	11.6 ± 8.1 (8.4–14.8)	10.5 ± 4.7 (8.7–12.2)	NS
Abdominal circumference (cm)	103.4 ± 16.5 (96.8–109.9)	103.3 ± 11.9 (98.9–107.8)	NS
Waist circumference (cm)	95.3 ± 15.3 (89.3–101.4)	96.1 ± 10.7 (92.1–100.1)	NS
Hip circumference (cm)	106.8 ± 14.6 (101.0–112.5)	105.6 ± 12.4 (100.9–110.2)	NS
Leg circumference (cm)	53.5 ± 7.0 (50.7–56.3)	53.8 ± 6.3 (51.4–56.2)	NS
WHR	0.89 ± 0.10 (0.86–0.93)	0.91 ± 0.09 (0.88–0.95)	NS
Total body fat (kg)	30.7 ± 14.8 (24.9–36.6)	30.4 ± 12.7 (25.6–35.1)	NS
Total body fat (%)	32.5 ± 10.4 (28.3–36.6)	34.3 ± 11.4 (30.0–38.5)	NS
Lean body mass (kg)	59.7 ± 10.2 (55.7–63.8)	55.9 ± 11.0 (51.8–60.0)	NS
Lean body mass (%)	67.5 ± 10.4 (63.4–71.6)	65.6 ± 11.4 (61.4–69.9)	NS

Data given as: *n* (%) or mean ± standard deviation (95% CI); SBP = systolic blood pressure; DBP = diastolic blood pressure; TC = total cholesterol; HDL = high-density lipoproteins; LDL = low-density lipoproteins; TGA = triglycerides; FPG = fasting plasma glucose; TSH = thyroid-stimulating hormone; PRL = prolactin; BMI = body mass index; FMI = fat mass index; WHR = waist-to-hip ratio; NS = non significant.

## Appendix

**Table A1:** Antipsychotic and Antidepressive Drugs in Study Group.

Sarcosine Group			Placebo Group		
Patient	Antipsychotic (*dose*, *mg*)	Antidepressant (*dose*, *mg*)	Patient	Antipsychotic (*dose*, *mg*)	Antidepressant (*dose*, *mg*)
1	Ari (*7.5*)	Ser (*50*)	1	Ola (*10*)	
2	Ari (*30*)	Ser (*25*)	2	Ola (*20*)	
3	Ari (*15*), Ola (*20*)		3	Ola (*20*)	
4	Ari (30), Ola (*5*)		4	Ari (*30*)	
5	Ola (*10*)	Ser (*50*)	5	Ari (*30*)	
6	Ola (*2.5*)	Ser (*50*)	6	Ola (*5*)	Ser (*50*)
7	Ami (*600*), Que (*25*)		7	Ola (*20*)	Ser (*200*)
8	Ami (*100*), Que (*400*)		8	Ami (*200*), Que (*150*)	
9	Ris (*4*)	Cit (*30*)	9	Ami (*400*), Que (*100*)	
10	Ris (*5.5*)	Cit (*20*)	10	Ari (*30*), Que (*100*)	
11	Ola (*10*)		11	Ari (*30*), Que (*25*)	
12	Que (*800*)		12	Flp (*200/3 weeks* *)	
13	Sul (*500*)		13	Ami (*400*), Ola (*20*)	
14	Per (*300*)		14	Ari (*7.5*), Ola (*12.5*)	
15	Ami (*600*), Ola (*20*)		15	Flp (*3*), Ola (*20*)	
16	Ola (*10*), Ris (*3*)		16	Per (*125*), Ris (*50/2 weeks **)	
17	Ola (*10*), Sul (*400*)		17	Que (*200*), Ris (*4*)	
18	Ola (*25*)	Esc (*10*)	18	Que (*700*), Zuc (*300/2 weeks* *)	
19	Que (*400*), Ris (*50/2 weeks* *)		19	Ami (*200*)	Ven (*225*)
20	Ari (*30*), Flp (*6*)		20	Flp (*200/4 weeks* *), Ris (*0.5*)	
21	Ami (*600*), Pro (*300*)		21	Sul (*50*)	Cit (*60*)
22	Ami (*300*)	Fvx (*100*)	22	Flp (*3*)	Cit (*20*)
23	Flp (*6*), Per (*300*)		23	Ami (*600*), Ola (*10*)	Cit (*10*)
24	Ami (*800*), Ola (*20*), Que (*200*)		24	Ari (*30*), Que (*600*)	Cit (*20*)
25	Ami (*400*), Ola (*5*)	Flx (*10*)	25	Ami (*400*), Hal (*3*), Que (*400*)	
26	Ari (*30*), Ola (*15*)	Ser (*100*)	26	Lev (*50*), Que (*200*)	Ser (*50*)
27	Ola (*15*), Ami (*200*)	Ser (*200*)	27	Ari (*30*), Ris (*2.5*)	Fvx (*50*)
28	Ari (*15*)	Flx (*20*), Clo (*150*)	28	Ami (*600*), Pro (*100*)	Ven (*225*)
			29	Hal (*6*), Per (*300*), Sul (*800*)	
			30	Per (*250*), Zip (*160*)	Ser (*50*)

Antipsychotics: Ami, amisulpride; Ari, aripiprazole; Flp, flupenthixol; Hal, haloperidol; Lev, levomepromazine; Ola, olanzapine; Per, perazine; Pro, promazine; Que, quetiapine; Ris, risperidone; Sul, sulpiride; Zip, ziprasidone; Zuc, zuclopenthixol; * long-acting injection. Antidepressants: Cit, citalopram; Clo, clomipramine; Esc, escitalopram; Flx, fluoxetine; Fvx, fluvoxamine; Ser, sertraline; Ven, venlafaxine.
